# *Pontibacter diazotrophicus* sp. nov., a Novel Nitrogen-Fixing Bacterium of the Family *Cytophagaceae*

**DOI:** 10.1371/journal.pone.0092294

**Published:** 2014-03-19

**Authors:** Linghua Xu, Xian-Chun Zeng, Yao Nie, Xuesong Luo, Enmin Zhou, Lingli Zhou, Yunfan Pan, Wenjun Li

**Affiliations:** 1 State Key Laboratory of Biogeology and Environmental Geology & Department of Biological Science and Technology, School of Environmental Studies, China University of Geosciences (Wuhan), Wuhan, China; 2 School of Chemical and Material Engineering, Hubei Polytechnic University, Huangshi, China; 3 State Key Laboratory of Agricultural Microbiology, Huazhong Agricultural University, Wuhan, China; 4 Key Laboratory of Microbial Diversity in Southwest China, Ministry of Education, and Laboratory for Conservation and Utilization of Bio-resources, Yunnan Institute of Microbiology, Yunnan University, Kunming, China; Belgian Nuclear Research Centre SCK/CEN, Belgium

## Abstract

Few diazotrophs have been found to belong to the family *Cytophagaceae* so far. In the present study, a Gram-negative, rod-shaped bacterium that forms red colonies, was isolated from sands of the Takalamakan desert. It was designated H4X^T^. Phylogenetic and biochemical analysis indicated that the isolate is a new species of the genus *Pontibacter*. The 16S rRNA gene of H4X^T^ displays 94.2–96.8% sequence similarities to those of other strains in *Pontibacter*. The major respiratory quinone is menaquinone-7 (MK-7). The DNA G+C content is 46.6 mol%. The major cellular fatty acids are iso-C_15∶0_, C_16∶1_ω5c, summed feature 3 (containing C_16∶1_ω6c and/or C_16∶1_ω7c) and summed feature 4 (comprising anteiso-C_17∶1_B and/or iso-C_17∶1_I). The major polar lipids are phosphatidylethanolamine (PE), one aminophospholipid (APL) and some unknown phospholipids (PLs). It is interesting to see that this bacterium can grow very well in a nitrogen-free medium. PCR amplification suggested that the bacterium possesses at least one type of nitrogenase gene. Acetylene reduction assay showed that H4X^T^ actually possesses nitrogen-fixing activity. Therefore, it can be concluded that H4X^T^ is a new diazotroph. We thus referred it to as *Pontibacter diazotrophicus* sp. nov. The type strain is H4X^T^ ( = CCTCC AB 2013049^T^ = NRRL B-59974^T^).

## Introduction

The Takalamakan desert is situated in the middle of the Tarim basin, Xinjiang province of China. It is the world's second largest shifting sand desert. Taklamakan has another name “the Sea of Death” due to its extremely rigorous climate. The highest temperature reached 65.6°C in summer and the lowest was below −20°C in winter. Diurnal temperature difference reaches over 40°C. It is very arid in the Takalamakan area. The annual precipitation is less than 100 mm, while evaporation reaches 2500–3400 mm. Moreover, there are only trace-level organic compounds in the sands and soils. Although the environmental conditions are extremely rigorous, some plants, such as *Populus euphratica*, still exist in Taklamakan Desert [1].

It was shown that the oligotrophic ecosystem is largely dependent on nitrogen input from biological nitrogen fixation. Nitrogen-fixing bacteria are the only organisms capable of converting molecular N_2_ into NH_4_^+^, a more readily assimilated form of dissolved nitrogen [2]. Diazotrophic bacteria also play a vital role in stabilizing soil against erosion and altering the hydrological properties of crust-covered soils for the plants in the deserts of India, Israel, Morocco, Chile and China [3]–[8]. Nitrogen-fixing bacteria are thus important for maintaining the ecological equilibrium of deserts and improving the environment. However, few of diazotrophs have been isolated from the Takalamakan desert so far.

We described a novel nitrogen-fixing bacterium H4X^T^ isolated from Taklamakan Desert. We showed that this bacterium is a new species of the genus *Pontibacter*. The bacterium is able to grow very well in a nitrogen-free medium. We also found that this bacterium contains a typical nitrogen-fixing gene *nifH*. Acetylene reduction assay showed that H4X^T^ actually possesses nitrogen-fixing ability. Therefore, the isolate is a new diazotroph. The bacterium was thus referred to as *Pontibacter diazotrophicus* sp. nov. This is the first nitrogen-fixing bacterium isolated from Taklamakan Desert.

## Materials and Methods

### Ethics statement

No specific permits were required for the described field studies. We would like to confirm that the location is not privately-owned or protected in any way, and the field studies did not involve endangered or protected species.

### Isolation of diazotrophic bacteria

About 1.0 gram of sands were taken from a dune ridge of Taklimakan Desert (84.173400W, 40.485143N). Scattered grass can be seen at the sampling site. The sands were suspended in 0.85% (w/v) NaCl solution. After removal of insoluble sands and large particles, supernatant containing bacteria was serially diluted and plated onto an agar plate containing 1 g K_2_HPO_4_, 0.2 g MgSO_4_, 1 g CaCO_3_, 0.2 g NaCl, 5 mg FeSO_4_, 10 g glucose per liter (pH 7.0). The plate was incubated at 30°C for 2 weeks.

### 16S rRNA gene sequence analysis

Genomic DNAs of bacteria were isolated using MiniBEST Bacterial Genomic DNA Extraction Kit Version 2.0 (TaKaRa Biotechnology Co., Tokyo, Japan). 16S rRNA gene was amplified by PCR using the primers 27F and 1492R as described previously [9]. PCR products were gel purified and sequenced by Genscript (Nanjing, China). Pairwise sequence identities of 16S rRNA genes were calculated using the Eztaxon-e server (http://eztaxon-e.ezbiocloud.net/) [10]. Multiple sequence alignment was performed using ClustalW [11]. Phylogenetic trees were constructed using the maximum-likelihood and Bayesian method implemented in MEGA 5.0 and MrBayes v3.1, respectively [12], [13]. The topology of the tree was evaluated using the bootstrap resampling method with 1000 replicates.

### Phenotypic analysis

Bacterial morphology and motility were observed under a phase contrast microscope using the cells that were grown in the 0.3×Marine Broth 2216 (Difco) medium at 28°C into exponential phase. Gram staining was performed as described previously [14]. Salt tolerance was determined by growing the bacteria in 0.3×Marine Broth 2216 containing different concentrations of NaCl (0–10%, w/v), respectively. Bacterial growth at different temperatures (4, 10, 20, 28, 30, 35, 37, 42°C) and different pH values (5.0–11.0) were also examined. Oxidase activity was determined from the oxidation of 1% p-aminodimethylaniline oxalate. Catalase activity was tested by measuring bubble production after the application of 3% (v/v) hydrogen peroxide solution. Capability to hydrolyze starch (1%, w/v), cellulose (0.1%, w/v), chitin from crab shells (1%, w/v), casein (1%, w/v) and tyrosine (0.5%, w/v) were also tested as described previously [14]. Other enzyme activities and biochemical features were determined using the API kits (API 20NE, API 20E, API 50CH and API ZYM) according to the manufacturer's instruction (BioMerieux, France). DNA G+C content of the strain H4X^T^ was determined using HPLC (UltiMate 3000, Dionex) [15], [16]. Respiratory quinones were extracted and detected by HPLC as described previously [17]. Polar lipids were isolated using a standard TLC technique [18]. For analysis of fatty acid methyl esters (FAMEs), the isolate and closely related type strains from the genus *Pontibacter* were cultured on the 0.3×Marine Broth 2216 agar plate for appropriate time, respectively. FAMEs were further prepared and analyzed using Sherlock Microbial Identification System (MIDI, Inc., Newwark, USA).

### Nitrogen-free growth assay

Bacteria were initially grown in the 0.3×Marine Broth 2216 medium into exponential phase. Cells were harvested by centrifugation (8000 rpm, 10 min, JA 20 rotor, Beckman). The pellets were washed twice with 0.85% (w/v) NaCl solution, and re-suspended in distilled water. The suspension was inoculated into a nitrogen-free agar plate containing 19.45 g NaCl, 8.8 g MgCl_2_, 3.24 g Na_2_SO_4_, 1.8 g CaCl_2_, 0.55 g KCl, 0.16 g NaHCO_3_, 0.1 g Ferric citrate, 0.08 g KBr, 0.034 g SrCl_2_, 0.022 g H_3_BO_3_, 8.0 mg Na_2_HPO_4_, 4.0 mg Na_2_SiO_3_, 2.4 mg NaF per liter (pH 7.4). Survived bacteria were passaged at least 20 times on the agar plate. The strains *Azospirillum lipoferum* Sp59^T^ and *Escherichia coli* DH5α were included as positive and negative control, respectively.

### Measurement of nitrogenase activity

Bacterial nitrogenase activity of the strain H4X^T^ was examined using the acetylene reduction assay. The strains *Azospirillum lipoferum* Sp59^T^ and *Escherichia coli* DH5α were included as positive and negative control, respectively. Other members of the genus *Pontibacter*, such as *P*. *actinarum* KMM 6156^T^, *P*. *korlensis* X14-1^T^ and *P*. *xinjiangensis* 311-10^T^ were also included as parallel comparison. Bacteria were grown in the 0.3×Marine Broth 2216 medium into exponential phase at 28°C with shaking. Cells were harvested by centrifugation (8000 rpm, 10 min, JA 20 rotor, Beckman), and washed twice with 0.85% (w/v) NaCl solution. The cells were re-suspended in distilled water. Aliquots of 0.2 ml were inoculated into vials (21 ml) containing 10 ml of semisolid NFb medium [19]. Cultures were incubated, unshaken, at 28°C. After 48 hours, the vials were sealed with rubber stoppers. The gas phase in the headspace was replaced with acetylene (10% v/v). Ethylene content was measured at 13 h intervals. Measurement was performed using a gas chromatograph (GC-4000, GL Science inc., Tokyo, Japan) with a flame-ionization detector and a column (2.0 m×2.0 mm i.d., stainless steel) packed with GDX-502. Controls with medium and inoculated culture without acetylene gas were run in parallel to each strain for the full incubation time.

### *NifH* gene sequence analysis

Genomic sequence of the *nifH* gene was amplified by direct PCR followed by nested PCR using two pairs of primers FGPH19 and PolR (for direct PCR), PolF and AQER (for nested PCR) as described previously [20], [21]. The primers was designed to amplify the partial sequence of the *nifH* gene that codes for the amino acid sequence from residue 38 to 149 in nitrogenase H. The PCR products were gel purified and cloned into the pMD18-T^®^ vector (TaKaRa Biotechnology Co., Tokyo, Japan). Positive clones were sequenced by Genscript (Nanjing, China). Multiple sequence alignment of the deduced amino acid sequences of the *nifH* genes from the strain H4X^T^ and other closely related bacteria were performed using ClustalW [11]. Phylogenetic tree was constructed using the maximum-likelihood or Bayesian method [12], [13]. The topology of the tree was evaluated using the bootstrap resampling method with 1000 replicates.

## Results

### Isolation of candidate diazotrophic bacteria from Taklamakan Desert

From 1.0 g of sands, we isolated twenty-six different bacteria that are capable of growing well in the nitrogen-free medium. Among them, twenty-five isolates formed white or whitish colonies, and the last one formed red colonies. Sequence analysis for the 16S rRNA genes of these bacteria showed that we discovered a new strain of bacteria with potential nitrogen-fixing activity, which was designated H4X^T^.

### Phylogeny of 16S rRNA gene sequences

**Figure 1:**
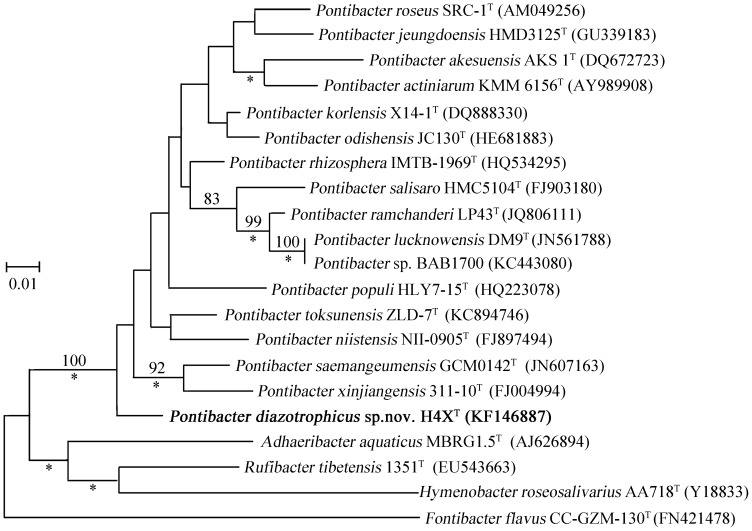
Maximum-likelihood phylogenetic tree based on multiple sequence alignment of 16S rRNA genes of the isolate *Pontibacter diazotrophicus* sp. nov. H4X^T^ and other closely related type strains. Bootstrap values (expressed as percentages of 1000 replicates) that are >75% are shown at branch points. Asterisks indicate that the corresponding nodes were also recovered in the Bayesian tree. Bar, 0.01 substitutions per nucleotide position.

The 16S rRNA gene sequence of H4X^T^ shows 96.8% and 95.5% identities to those of *Pontibacter toksunensis* and *Pontibacter saemangeumensis*, respectively. It also shows 94.2–95.4% identities to those of other species of the genus *Pontibacter*, such as *P*. *korlensis*, *P*. *lucknowensis*, *P*. *actiniarum*, *P*. *ramchanderi*, *P*. *odishensis*, *P*. *roseus*, *P*. *xinjiangensis*, *P*. *akesuensis*, *P*. *niistensis*, *P*. *populi*, *P*. *rhizosphera*, *P*. *salisaro*, *P*. *indicus* and *P*. *jeungdoensis*. Phylogenetic analysis indicated that the strain H4X^T^ is most closely related with *P*. *saemangeumensis* GCM0142^T^ and *P*. *xinjiangensis* 311-10^T^ (Figure 1). Thus, it is likely that H4X^T^ represents a new species of the genus *Pontibacter*.

### Chemotaxonomic characterization

**Figure 2:**
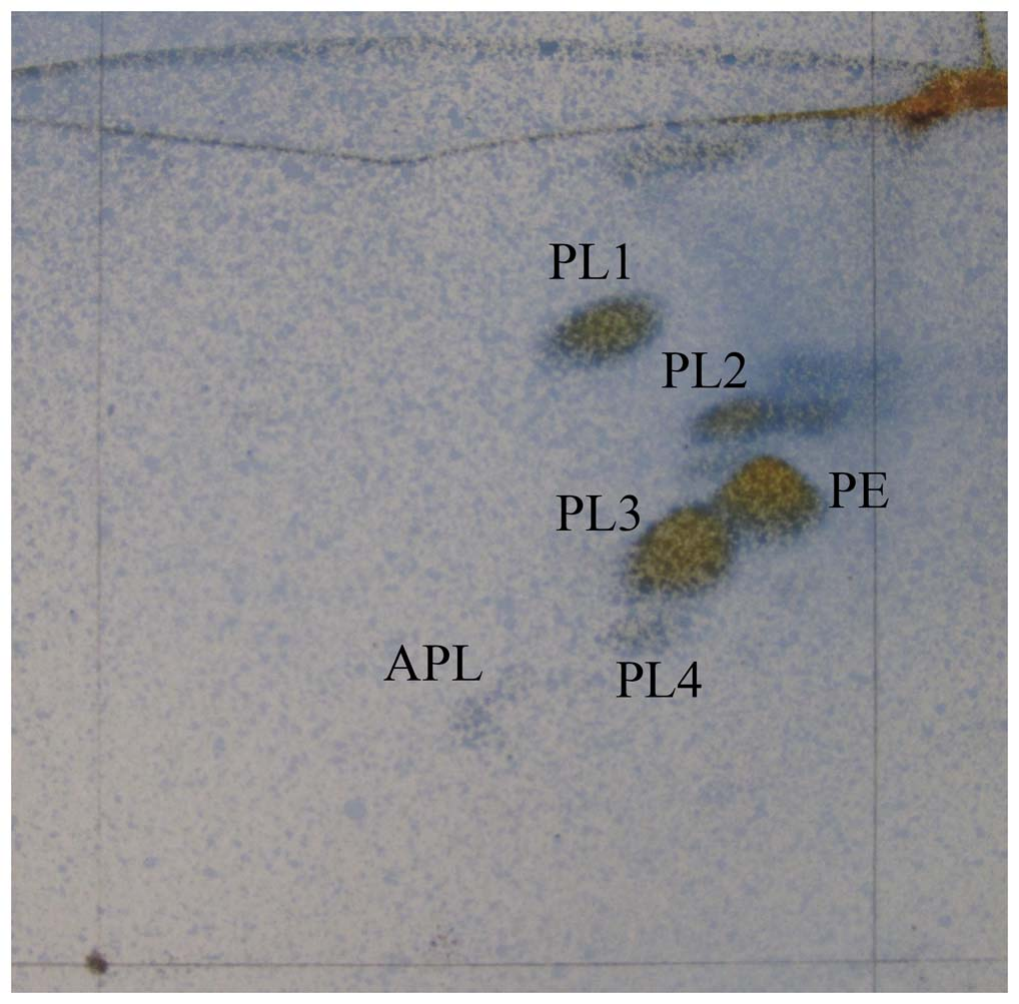
Polar lipids of the strain *Pontibacter diazotrophicus* sp. nov. H4X^T^. PE, phosphatidylethanolamine; APL, aminophospholipid; PL1-4, unknown phospholipids.

The G+C content of H4X^T^ was 46.6 mol%, which falls within the range for the genus *Pontibacter*. Only menaquinone-7 (MK-7) was detectable as respiratory menaquinone. Phosphatidylethanolamine was found to be one of the major polar lipids in the cells. In addition, we found that there are several unknown phospholipids and an aminophospholipid (Figure 2). The major fatty acids include iso-C_15∶0_ (10.9%), C_16∶1_ω5c (14.3%), summed feature 3 (containing C_16∶1_ω6c and/or C_16∶1_ω7c) (21.6%) and summed feature 4 (comprising anteiso-C_17∶1_ B and/or iso-C_17∶1_ I) (31.9%) (Table 1). All these chemotaxonomic properties of the strain H4X^T^ are consistent with those of other members of the genus *Pontibacter* described so far [22], [23].

**Table 1:** Fatty acid profiles (%) of the strain H4X^T^ and related type strains of the genus *Pontibacter*.

Fatty acid	1	2	3	4	5[^25^] ^*^
C_14:0_	tr				
iso-C_13:0_ 3OH			tr	1.3	
Summed feature 1	1.7	tr	1.5	tr	
iso-C_15:0_	10.9	14.8	35.1	9.3	22.3
anteiso-C_15:0_		tr		1.7	2.5
C_15:1_ ω6c	1.1				2.9
iso-C_16:1_ H	tr	tr		1.0	3.5
iso-C_16:0_		tr	tr	tr	1.1
Summed feature 3	21.6	7.5	10.2	23.0	6.2
C_16:1_ω5*c*	14.3	6.5	3.1	10.4	4.1
C_16:0_	2.4	2.9	1.8	2.1	tr
iso-C_15:0_ 3OH	1.4	3.8	3.5	2.7	1.2
iso-C_15:1_ F				tr	
Summed feature 9	tr	tr		1.5	
Summed feature 4	31.9	31.5	25.8	26.4	36.8
iso-C_17:0_	1.8	5.6	3.7	1.4	1.5
anteiso-C_17:0_				tr	
C_17:0_		tr			
C_17:1_ *ω*6*c*	2.6	3.5	1.4	2.1	4.3
Summed feature 5		1.4	1.3	1.3	tr
Summed feature 8	1.4	tr		1.9	tr
C_18∶0_	tr	1.0	1.2	1.8	tr
iso-C_17∶0_ 3OH	5.1	10.5	8.6	7.6	3.9
iso-C_18∶1_ H		tr			
iso-C_19∶1_ I		3.1			
C_20∶0_				1.1	

Strains: 1, H4X^T^; 2, *Pontibacter korlensis* X14-1^T^; 3, *Pontibacter actiniarum* KMM 6156^T^; 4, *Pontibacter xinjiangensis* 311-10^T^; 5, *Pontibacter saemangeumensis* GCM0142^T^. All data were taken from this study, otherwise it was indicated by asterisks. tr, trace (<1%).

Summed feature 1: C_15∶1_ iso H/C_13∶0_ 3OH.

Summed feature 3: C_16∶1_ ω6c/C_16∶1_ω7c.

Summed feature 4: C_17∶1_ anteiso B/iso I.

Summed feature 5: C_18∶2_ω6, 9c/C_18∶0_ ante.

Summed feature 8: C_18∶1_ω7c/C_18∶1_ω6c.

Summed feature 9: iso-C_17∶1_ω9c/C_16∶0_ 10 methyl.

### Phenotypic features

The bacterial cells of the strain H4X^T^ are Gram-staining negative, mobile by gliding. Typical cells are straight, slightly curved or curved rods. The bacteria form red colonies on the 0.3×Marine Broth 2216 agar plate. Colonies are convex and circular with entire margin. The cells are catalase-positive and oxidase-positive (Table 2). The strain grows at a wide range of temperatures from 4°C to 40°C, and the optimum is 30°C. Growth occurs at pH values of 6.0–8.0, and the optimum pH is 7.0. The strain tolerates high salt concentrations up to 8% (w/v) NaCl. We found that there are a lot of phenotypic features of the strain H4X^T^ that make it distinguishable from the reference species (Table 2). These data suggest that the strain H4X^T^ represents a novel species of the genus *Pontibacter*.

**Table 2:** Differential properties of the strain H4X^T^ and related type strains of the genus *Pontibacter.*

Characteristic	1	2	3	4	5[^25^]
G+C concent (%)	46.6	48.2*	48.7*	47.8*	48.9*
growth temperature range (°C)	4–40	7–45*	6–43*	4–37*	5–30*
pH	6.0–8.0	5.5–11.0*	nd	6.0–10.0*	6.0–10.0*
salinity (%)	0–8	0–8*	0–10*	0–5*	0–2*
motility	+	+	+	−	−*
oxidase	+	−	−	−	+*
nitrate reduction	−	−	−	+	−*
**Hydrolysis of**					
starch	+	+	−	+	+*
casein	+	−	−	+	+*
gelatin	−	+	+	+	−*
aesculin	+	+	−	+	+*
ONPG	+	w	−	w	+*
**Assimilation(20NE/32GN)**					
D-glucose	w	+	−	−	nd
L-arabinose	w	−	−	−	nd
D-mannose	+	+	−	−	nd
D-mannitol	+	−	−	−	nd
N-acetyl-glucosamine	+	+	−	−	nd
D-maltose	+	+	−	−	nd
gluconate	+	w	−	−	nd
adipate	w	−	−	−	nd
malate	w	−	−	−	nd
trisodium citrate	+	−	−	−	nd
phenylacetic acid	w	−	−	−	nd
L-rhamnose	−	w	−	−	nd
D-ribose	+	−	−	−	nd
inositol	w	+	−	−	nd
D-saccharose	+	+	−	−	nd
suberic acid	−	+	−	−	nd
malonate	−	w	−	−	nd
lactate	+	−	−	−	nd
L-alanine	w	+	−	−	nd
5-ketogluconate	w	+	−	−	nd
glycogen	w	+	−	−	nd
D-melibiose	−	+	−	−	nd
capric acid	+	w	−	−	nd
L-histidine	−	+	−	−	nd
2-ketogluconate	−	+	−	−	nd
3-hydroxybutyric acid	+	+	−	−	nd
**Enzymes(ZYM)**					
lipase(C14)	−	+	−	−	−*
cystinearylamidase	w	+	+	+	−*
trypsin	+	−	+	+	+*
*α*-chymotrypsin	−	−	−	+	−*
*α*-galactosidase	−	+	−	+	+*
*β*-galactosidase	−	+	−	+	+*
*β*-glucuronidase	−	+	−	−	−*
*α*-glucosidase	−	+	+	−	+*
*β*-glucosidase	−	+	−	−	+*
*α*-mannosidase	−	−	−	−	+*

Strains: 1, H4X^T^; 2, *P*. *korlensis* X14-1^T^; 3, *P*. *actiniarum* KMM 6156^T^; 4, *P*. *xinjiangensis* 311-10^T^; 5, *P*. *saemangeumensis* GCM0142^T^. All data were obtained from this study unless otherwise indicated by asterisks. All strains produce N-acetyl-β-glucosaminidase, naphthol-AS-BI-phosphohydrolase, acid phosphatase, leucinearylamidase, valine arylamidase, esterase lipase (C8), alkaline phosphatase and esterase(C4); all strains are negative for hydrolysis of tyrosine, chitin and cellulose, production of indole and H_2_S, V-P test, glucose fermentation, and activities of arginine dihydrolase, urease, lysine decarboxilase, ornithine decarboxilase, tryptophane deaminase and α-fucosidase. The strains H4X^T^, *P*. *korlensis* X14-1^T^, *P*. *actiniarum* KMM 6156^T^ and *P*. *xinjiangensis* 311-10^T^ are not able to assimilate itaconic acid, acetate, L-serine, salicin, L-fucose, D-sorbitol, propionate, valeric acid, 3-hydroxybenzoic acid, 4-hydroxybenzoic acid and L-proline.

+: positive; -: negative; w: weakly positive; nd: not determined.

### Nitrogen-fixing properties

The cells of the strains H4X^T^, *E*. *coli* DH5α, *A*. *lipoferum* Sp59^T^, *P*. *actinarum* KMM 6156^T^, *P*. *korlensis* X14-1^T^ and *P*. *xinjiangensis* 311-10^T^ were inoculated onto the agar plates containing nitrogen-free medium, and passaged at least 20 times, respectively. We found that only H4X^T^ and the positive control *A*. *lipoferum* Sp59^T^ were capable of proliferating in the nitrogen-free medium even after multiple passages. Thus, it is likely that the isolate is a diazotroph.

The discovery that the nitrogenase enzyme responsible for nitrogen-fixation also reduced acetylene to ethylene provided a useful assay for the quantification of the nitrogen-fixation process [24]. To further confirm that H4X^T^ is a nitrogen-fixer, we performed acetylene reduction assay. As shown in Table 3, we found that, if the assay for the strain H4X^T^ was performed without acetylene, ethylene was not detectable. This suggests that the strain H4X^T^ does not produce detectable native ethylene. The strain H4X^T^ was able to convert acetylene into ethylene at the rate of 7.13±1.2 nmol per hour per 10^8^ cells at 28°C, whereas the positive control, *A*. *lipoferum* Sp59^T^ can reduce ethylene at the rate of 97.85±1.6 nmol per hour per 10^8^ cells. However, the negative control, *E*. *coli* DH5α, and other members of the genus *Pontibacter*, such as *P*. *actinarum* KMM 6156^T^, *P*. *korlensis* X14-1^T^ and *P*. *xinjiangensis* 311-10^T^ were totally unable to reduce acetylene.

**Table 3:** Nitrogenase activity detected with acetylene reduction assay.

Strains	Acetylene reduction activity^*^ (nmol C_2_H_4_ per hour per 10^8^ cells)	
	Culture without C_2_H_2_	Culture with C_2_H_2_
*Pontibacter diazotrophicus* sp. nov. H4X^T^	0	7.13±1.2
*Azospirillum lipoferum* Sp59^T^	0	97.85±1.6
*Escherichia coli* DH5α	0	0
*Pontibacter actinarum* KMM 6156 ^T^	0	0
*Pontibacter xinjiangensis* 311-10^T^	0	0
*Pontibacter korlensis* X14-1^T^	0	0

*Data were obtained from three independent experiments.

**Figure 3:**
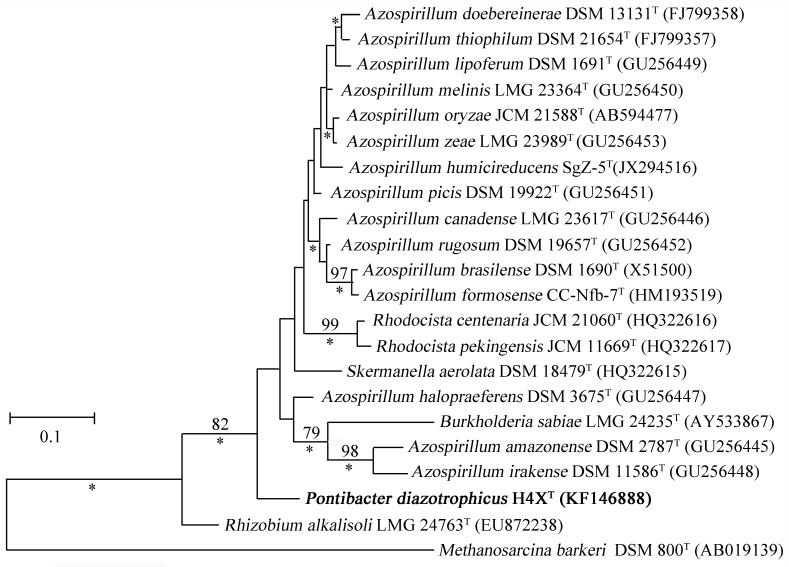
Maximum-likelihood tree based on multiple sequence alignment of the *nifH* genes from *Pontibacter diazotrophicus* sp. nov. H4X^T^ and other closely related bacterial strains. Bootstrap values (expressed as percentages of 1000 replicates) that are >75% are shown at branch points. Asterisks indicate that the corresponding nodes were also recovered in the Bayesian tree. Bar, 0.1 substitutions per nucleotide position.

Moreover, we detected the existence of a nitrogenase gene (*nifH*) in the stain H4X^T^. The *nif* genes are a family of genes encoding enzymes involved in the fixation of atmospheric nitrogen. PCR strategy was employed to amplify the *nifH* gene from the genomic DNAs of H4X^T^ using two pairs of primers FGPH19 and PolR, PolF and AQER as described previously [20], [21]. The PCR amplification using the primers FGPH19 and PolR yielded some non-specific bands. Nested PCR using the primers PolF and AQER was further employed to increase the specificity of DNA amplification. PCR products were gel purified and cloned into a T-vector for sequencing. The result showed that we successfully obtained the partial genomic sequence (298 bp) of the *nifH* gene from the strain H4X^T^, but failed to get it from other related species of the genus *Pontibacter*, including *P*. *actiniarum* KMM 6156^T^, *P*. *korlensis* X14-1^T^ and *P*. *xinjiangensis* 311-10^T^. Phylogenetic analyses indicated that the *nifH* gene of the strain H4X^T^ is most closely related to those of some species of the genus *Azospirillum*, including *A*. *halopraeferens*, *A*. *picis* and *A*. *rugosum* (Figure 3).

Therefore, based on the phenotypic, genotypic and biochemical properties of the strain H4X^T^, it can be concluded that this bacterium represents a novel species of the genus *Pontibacter*. It was thus referred to as *Pontibacter diazotrophicus* sp. nov. It is noteworthy that *P*. *diazotrophicu*s is the first nitrogen fixer described so far from the genus *Pontibacter*.

### Description of Pontibacterdiazotrophicus sp. nov

*Pontibacterdiazotrophicus* (di.a.zo.tro'phi.cus. Gr.prefix di, two, double; N.L.n. azotum, nitrogen; Gr.adj.trophikos, nursing, ending or feeding; M.L. masc. adj. *diazotrophicus*, one that feeds on dinitrogen).

Cells are Gram-staining negative, rod-shaped (0.4–0.6×1.2–2.0 μm) and mobile by sliding. They form circular, convex, and red colonies with entire margin on the 0.3×Marine Broth 2216 agar plate. Growth occurs at temperatures from 4 to 40°C (optimum 30°C), at pH 6.0–8.0. The isolate grows in 0–8% (w/v) NaCl. The isolate is oxidase positive and catalase positive. It possesses the *nifH* gene, and is capable of fixing nitrogen. It can hydrolyse starch, casein, aesculin and ONPG, but not gelatin, tyrosine, chitin and cellulose. It is negative for nitrate reduction, H_2_S production, V-P test, indole production and glucose acidification. It assimilates D-mannose, D-mannitol, N-acetyl-glucosamine, D-maltose, gluconate, trisodium citrate, D-ribose, D-saccharose, lactate, capric acid and 3-hydroxybutyric acid, but not L-rhamnose, suberic acid, malonate, D-melibiose, L-histidine, 2-ketogluconate, itaconic acid, acetate, 3-hydroxybenzoic acid, L-serine, salicin, L-fucose, D-sorbitol, propionate, valeric acid, 4-hydroxybenzoic acid and L-proline in the API 20NE and API 32GN system. It also has the activities of alkaline phosphatase, esterase (C4), esterase lipase (C8), leucinearylamidase, valine arylamidase, cystinearylamidase, trypsin, acid phosphatase, naphthol-AS-BI-phosphohydrolase and N-acetyl-β-glucosaminidase, but negative for those of lipase (C14), α-chymotrypsin, *α*-mannosidase, *α*-fucosidase, *α*-galactosidase, *β*-galactosidase, *β*-glucuronidase, *α*-glucosidase, *β*-glucosidase, arginine dihydrolase, lysine decarboxilase, ornithine decarboxilase, tryptophane deaminase and urease. The major fatty acids are iso-C_15∶0_, C_16∶1_ω5*c*, summed feature 3 (containing C_16∶1_ω6c and/or C_16∶1_ω7c) and summed feature 4 (comprising anteiso-C_17∶1_ B and/or iso-C_17∶1_ I). MK-7 is the predominant menaquinone. The major polar lipids are composed of PE, APL and unknown phospholipids. The G+C content of the genomic DNA of the type strain is 46.6 mol%.

The type strain, H4X^T^ ( = CCTCC AB 2013049^T^ = NRRL B-59974^T^), was isolated from the sands of the Takalamakan desert.

## Discussion

The genus *Pontibacter*, first described by Nedashkovskaya et al., is a member of the family *Cytophagacea* [22]. Until now, at least fifteen species of this genus have been isolated from different habitats, including *P*. *actiniarum* and *P*. *saemangeumensis* from sea water [22], [25], *P*. *roseus* from occasional drainage system [26], *P*. *xinjiangensis*, *P*. *korlensis*, *P*. *toksunensis* and *P*. *akesuensis* from desert soils [8], [23], [27], [28], *P*. *niistensis* and *P*. *populi* from forest soil [29], [30], *P*. *rhizosphera* from the rhizosphere soil of Nerium indicum [31], *P*. *salisaro*, *P*. *jeungdoensis* and *P*. *odishensis* from solar saltern [32]–[34], and *P*. *lucknowensis* and *P*. *ramchanderi* from the hexachlorocyclohexane contaminated soil [35], [36]. Among all members of the family *Cytophagacea*, none has been found to have nitrogen-fixing activity so far. Our study showed that the strain H4X^T^ is capable of growing well in a nitrogen-free medium. We also found that it possesses the *nifH* gene potentially encoding nitrogenase. Acetylene reduction assay suggested that H4X^T^ possesses the nitrogenase activity. Therefore, H4X^T^ is actually a diazotroph. This is the first report of a nitrogen-fixing bacterium belonging to the genus *Pontibacter*. Until now, only a few of the bacterial strains belonging to *Cytophaga*-*Flavobacterium*-*Bacteroides* (CFB) group have been found to be diazotrophs [37]. Our study expands the knowledge of nitrogen-fixing bacteria in this evolutionary lineage.

It was shown that genes involved in nitrogen fixation may be transferred between distantly related species belonging to different phyla of bacteria [2], [38]. Lateral gene transfer plays a major role in the genome evolution of *Pontibacter* sp. [39]. Here, we found that the nucleotide sequence of the *nifH* gene of the strain H4X^T^ is closely related to those from *Azospirillum* sp., affiliated with *α*-*Proteobacteria*. Therefore, it is interesting to further explore whether the *nifH* gene of the strain H4X^T^ was acquired by horizontal gene transfer.

It was shown that bacteria inhabiting the oligotrophic Taklamakan desert could largely depend on the nitrogen input from biological nitrogen fixation. Thus, the *nifH* gene encoding the nitrogenase that is capable of converting molecular N_2_ into NH_4_^+^, could undergo high selective pressure. This would lead to high degree of sequence homology between the *nifH* gene of the strain H4X^T^ and those of other bacterial species from the desert.
